# Editorial: Epigenomic and epitranscriptomic basis of development and human disease

**DOI:** 10.3389/fcell.2023.1128745

**Published:** 2023-02-20

**Authors:** Shunliang Xu, Zhao-Qian Teng, Xuekun Li, Yujing Li

**Affiliations:** ^1^ Department of Neurology, The Second Hospital, Cheeloo College of Medicine, Shandong University, Jinan, China; ^2^ Institute of Zoology, Chinese Academy of Sciences (CAS), Beijing, China; ^3^ Institute of Translational Medicine, School of Medicine, Zhejiang University, Hangzhou, China; ^4^ Department of Human genetics, Emory University School of Medicine, Atlanta, GA, United States

**Keywords:** epigenomics, epitranscriptomics, development, human disease, base modifications, histone modifications

Dynamic and precise regulation of gene expression is orchestrated genetically and epigenetically in accordance with developmental stages and in response to environment stimuli. Aberrant genetic or epigenetic events could devastate gene expression, contributing to pathogenesis of disease. Epigenetic markers of base modifications such as 5-cytosine (5-mC), 5-hydroxycytosine (5-hmC) and adenosine methylation m^6^A have been appreciated as of the well-characterized and the most important epigenetic/epitranscriptomic markers essentially functional in almost all the biological process of mammals. The main components in the machine complexes responsible for generation and functions of these epi-markers have been characterized in recent years. These components display tissue-specific and delicate spatiotemporal patterns, and their fine-tuned orchestration contributes to the precise and dynamic regulation of the epigenomic and the epitranscriptomic landscapes, ensuring the normal growth, development, and reproduction. More importantly, the emerging evidence links the aberrant regulation of these epi-markers to pathogenesis of multiple diseases, potentially translatable to clinical applications for therapy.

This Research Topic mainly focuses on the epigenomic and epitranscriptomic basis of human diseases. The aim of the topic is to provide a broad overview of current research on epigenetic aspect of development and pathogenesis of human diseases.

Total 13 of articles published in this Research Topic focus on epigenetic regulation of learning and memory, immunological functions, and tumorigenesis. Two of them are related to base modification and human disease. Xu et al., found that deficiency in TET1, one of the erasers for base demethylation impairs learning and memory. In addition to neurological disorder, methylation status is also linked to cancers. One research article addresses the critical role of histone methyltransferase KMT5B on initiation of glioblastoma (GBM) *via* epigenetically regulating a subset of GBM-related genes associated with hypermethylation and 5-hmC loss of genomic DNA Lopez et al.

Histone modifications are associated with lipogenesis and obesity by repression or activation of gene expression. Two research articles are related to the link of histone modifications with metabolic disorders and autophagy involved in pulpitis. Wang et al. found that dynamic histone modifications could activate a subset of genes involved in lipogenesis, energy metabolism and inflammation under the high-fat diet (HFD) conditions, addressing the contribution of the eating habits to the related pathology *via* histone modification.

Autophagy is regulated epigenetically as well during development. Yin et al., screened several groups of writers and erasers for histone methylations under TNFα treatment, providing important information on the epigenetic regulation of autophagy genes during pulpitis, and more importantly this study could be translated to a novel clinical therapy.

As a summary, the research and review articles published in this Research Topic provide important scientific information in a convenient manner that is targeted toward readers with interest in the *epigenomic and epitranscriptomic basis of development and human diseases*.

